# Loneliness in elderly in the covid-19 pandemic

**DOI:** 10.1192/j.eurpsy.2022.683

**Published:** 2022-09-01

**Authors:** S. Suárez-Gómez, P. Rodrigues, A. Matos-Pires

**Affiliations:** Hospital José Joaquim Fernandes, Psychiatry, Beja, Portugal

**Keywords:** Covid-19, Elderly, Geriatric Psychiatry, Loneliness

## Abstract

**Introduction:**

Loneliness and social isolation condition the health of those over 65 years of age, increasing morbidity and mortality. The pandemic caused by Covid-19 has been a health emergency in which the negative effects have been increased by loneliness. We can define several types of loneliness: physical loneliness, moral loneliness and social isolation.

**Objectives:**

The objective was to analyze the impact of Covid-19 on the feeling of loneliness in those over 65 years of age during the last year of the pandemic.

**Methods:**

A bibliographic search was carried out in Pubmed with the terms “loneliness in elderly in the covid-19 pandemic” with the filters “abstract” and “in the last 1 year”, selecting the studies whose title included the terms “loneliness”, “elderly” or “older people” and “Covid-19 ” or “SARS-Cov-2”. The search gave rise to 13 results, of which the content of the abstracts was qualitatively analyzed.

**Results:**

All studies found an increase in loneliness in the elderly, and more than 50% reported a decrease in this feeling in the elderly trained in new technologies. Other aspects that stood out to influence were comorbidity, resilience, economic situation, social support and subjective feeling of vulnerability.

**Conclusions:**

Older adults avoid direct social contact to protect themselves. This may result in loneliness, that can have serious consequences in terms of morbidity and mortality. To mitigate loneliness they can use online social media, but older adults need to be trained. Institutions and public powers have the obligation to ensure individual and collective security, and protect the integrity of people from dangers.

**Disclosure:**

No significant relationships.

